# Effect of accessory hepatic artery reconstruction on prognosis in orthotopic liver transplantation: a single center experience

**DOI:** 10.1186/s12893-023-02021-7

**Published:** 2023-05-19

**Authors:** Rui Zhang, He-Zhao Zhang, Tian Han, Zhi-Gang Wei, Zhi-Yong Shi, Jun Xu

**Affiliations:** Department of Hepatobiliary and Pancreatic Surgery, Liver Transplantation Center, The First Hospital of Shanxi Medical University, No. 56 Xinjian South Road, Shanxi Province 030001 Taiyuan, China

**Keywords:** Accessory hepatic artery, Reconstruction, Orthotopic liver transplantation

## Abstract

**Background:**

In orthotopic liver transplantation (OLT), preserving an aberrant hepatic artery (AHA) can increase the number of arterial anastomoses and may lead to arterial-related complications. AHA includes accessory hepatic artery and replaced hepatic artery. Herein, the purpose of our research is to evaluate the requirement for accessory anastomosis in OLT.

**Methods:**

We retrospectively reviewed a total of 95 patients who underwent OLT in our hospital between April 2020 and December 2022. We found seven cases of donor livers with accessory HA. The method of arterial anastomosis and details of the diagnosis and treatment of complications were collated.

**Results:**

Among 95 consecutive patients with OLT, complications occurred in two of seven patients—patient 2 had an accessory right hepatic artery, while patient 5 had an accessory left hepatic artery. Patient 2 showed bile leakage leading to rupture and bleeding of the accessory HA anastomosis after OLT, and was treated with interventional coil embolization. In patient 5, hepatic artery thrombosis and accessory HA occlusion were treated with embolization and thrombolysis of the splenic artery and left gastric artery. During the intervention, we also found that the internal hepatic artery and accessory HA had communicating branches. After treatment, both patients remain healthy with no complications such as liver necrosis or liver abscess.

**Conclusion:**

An AHA can be ligated when assessed as an accessory artery. This can reduce the incidence of arterial complications, contribute to the perioperative management of liver transplantation (LT) patients, and improve the prognosis of LT.

## Introduction

The most common variations of the hepatic artery (HA) involve a left HA (LHA) originating from the left gastric artery (LGA), a right HA (RHA) arising from the superior mesenteric artery, or both. Aberrant LHAs (ALHAs) (or aberrant RHAs [ARHAs]) are defined as accessory if the left liver is vascularized by the main LHA, and as replaced when the arterial inflow is only from the ALHA. ALHAs or ARHAs can be either accessory or replaced. However, at the time of donor liver procurement, it is impossible to determine which of these two categories an anomalous artery belongs to because the intrahepatic branches cannot be explored [1].

During surgery, we often try to preserve an abberant HA (AHA) to ensure good blood supply to the liver and reduce the potential for postoperative liver necrosis and infection. However, preservation of an AHA will inevitably increase the number of arterial anastomosis or lead to increased arterial length or even angulation. In turn, this may increase the potential for postoperative anastomotic stenosis, thrombosis, aneurysm, rupture, and bleeding after surgery. Herein, we report the management of accessory HAs and the occurrence of postoperative complications in seven of 95 consecutive cases of orthotopic liver transplantation (OLT) in our transplantation center and evaluated the requirement for accessory HA anastomosis in OLT.

## Methods

From April 2020 to December 2022, 95 consecutive cases of whole-graft liver transplantations (LTs) were performed. Liver grafts were all procured from donors after brain death or cardiac death. We experienced seven patients with donor liver accessory HA. Complications related to arterial anastomosis occurred in two patients, which involved one patient with an accessory righe HA and one patient with an accessory left HA. We summarized the anatomical variants of the LT patients, as well as their postoperative complications, treatments, and outcomes. This study complied with the Helsinki conference and the Istanbul declaration, and was approved by the ethics committee of the first hospital of Shanxi Medical University on 13 January, 2023 (No. 21–2023).

### Patient 2

**Fig. 1:**
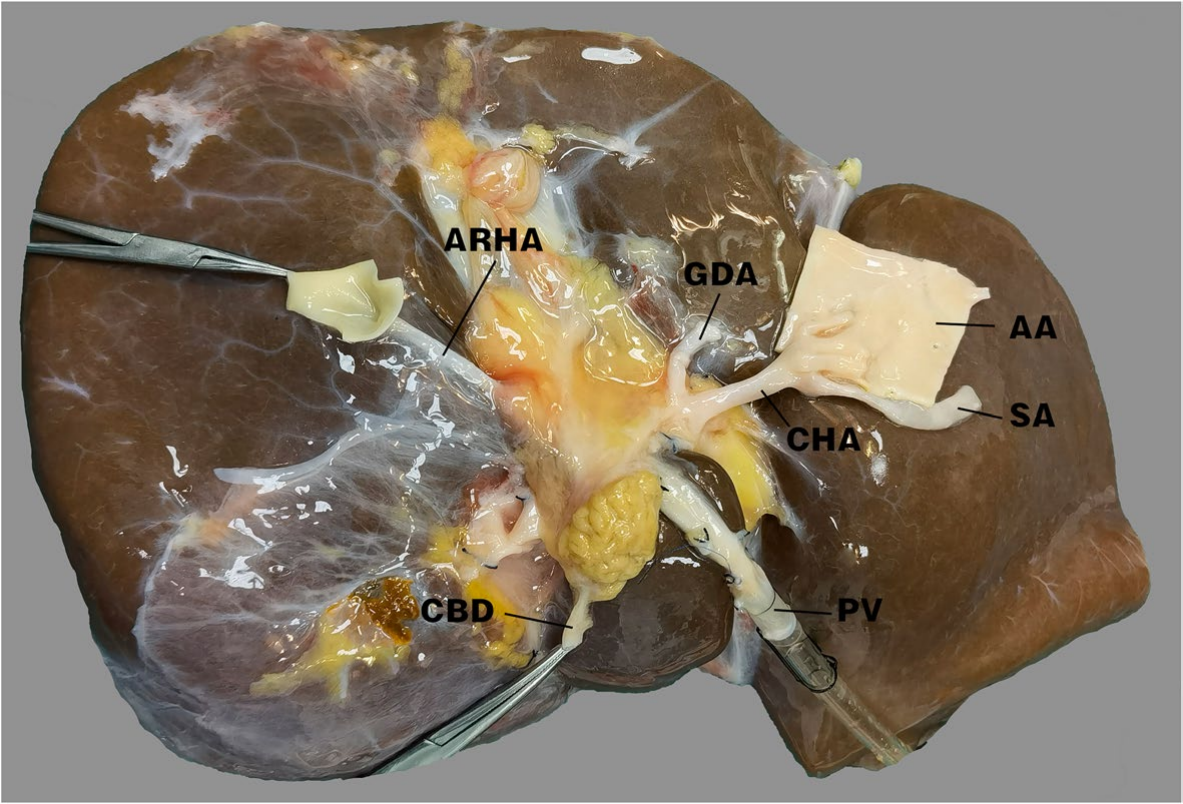
The accessory right hepatic artery originates from the superior mesenteric artery

The patient was male (56 years old) and underwent OLT in January 2021 because of Budd–Chiari syndrome complicated with portal vein thrombosis and cavernous transformation of the portal vein. The donor liver had an accessory righe HA from the superior mesenteric artery (Fig. 1). To ensure blood supply to the liver, the accessory right HA of the donor liver was anastomosed with the gastroduodenal artery (GDA) of the donor liver, while the donor celiac artery was anastomosed with the bifurcation of the recipient’s common HA and GDA. Because the anastomosed artery was too long to distort, it was padded straight with a gelatin sponge. Intraoperative Doppler ultrasonography showed good blood flow of the LHA and RHA.

### Patient 5

**Fig. 2:**
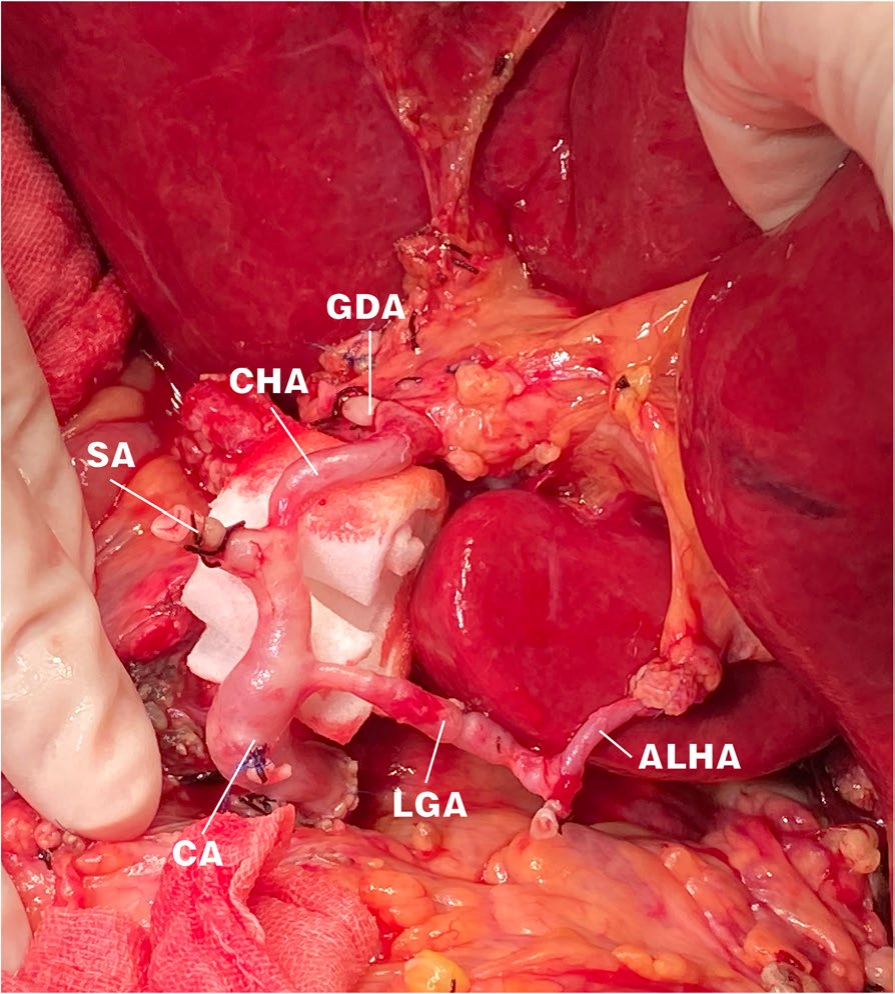
The accessory left hepatic artery originated from the left gastric artery. The celiac artery of the donor liver was anastomosed with the bifurcation of the gastroduodenal artery and the proper hepatic artery of the recipient. The long and tortuous hepatic artery was supported with a gelatin sponge

The patient was female (55 years old) and underwent OLT in July 2021 because of autoimmune cirrhosis and portal hypertension. The donor liver had an accessory left HA from the LGA. To maintain a complete liver blood supply, the celiac artery of the donor liver was anastomosed with the bifurcation of the recipient’s proper HA and GDA (Fig. 2). Because the anastomotic artery was long and tortuous, it was padded straight with a gelatin sponge. Intraoperative Doppler ultrasonography showed good blood flow of the LHA wand RHA.

## Results

A total of 95 OLT recipients (68 men, 27 women) aged between 27 and 68 years were included in this study. We experienced seven patients with a donor liver accessory HA. Complications occurred in two of these seven patients. Demographic and clinical data for the seven patients are summarized in Table 1.

**Table 1:** Demographic and clinical characteristics of seven patients who received accessory arterial reconstruction during orthotopic liver transplantation

Case	Gender	Age	Primary disease	MELD	Anatomical variations	Complications	Follow-up (Month)
1	M	53	HBV	18	ARHA branch SMA		31
2	M	56	Budd-Chiari syndrome	22	ARHA branch SMA	Rupture and bleeding of arterial anastomosis	24
3	M	36	HBV	16	ALHA branch LGA		24
4	F	57	PBC	24	ARHA branch SMA		20
5	F	55	PBC	19	ALHA branch LGA	Thrombosis of arterial anastomosis	17
6	M	45	HBV	22	ARHA branch SMA		16
7	M	58	HBV	16	ARHA branch SMA		15

*MELD* Model for end-stage liver disease, *HBV* Hepatitis B virus, *PBC* Primary biliary cirrhosis, *ARHA* Aberrant right hepatic artery, *SMA* Superior mesenteric artery, *ALHA* Aberrant left hepatic artery, *LGA* Left gastric artery

### Patient 2

**Fig. 3:**
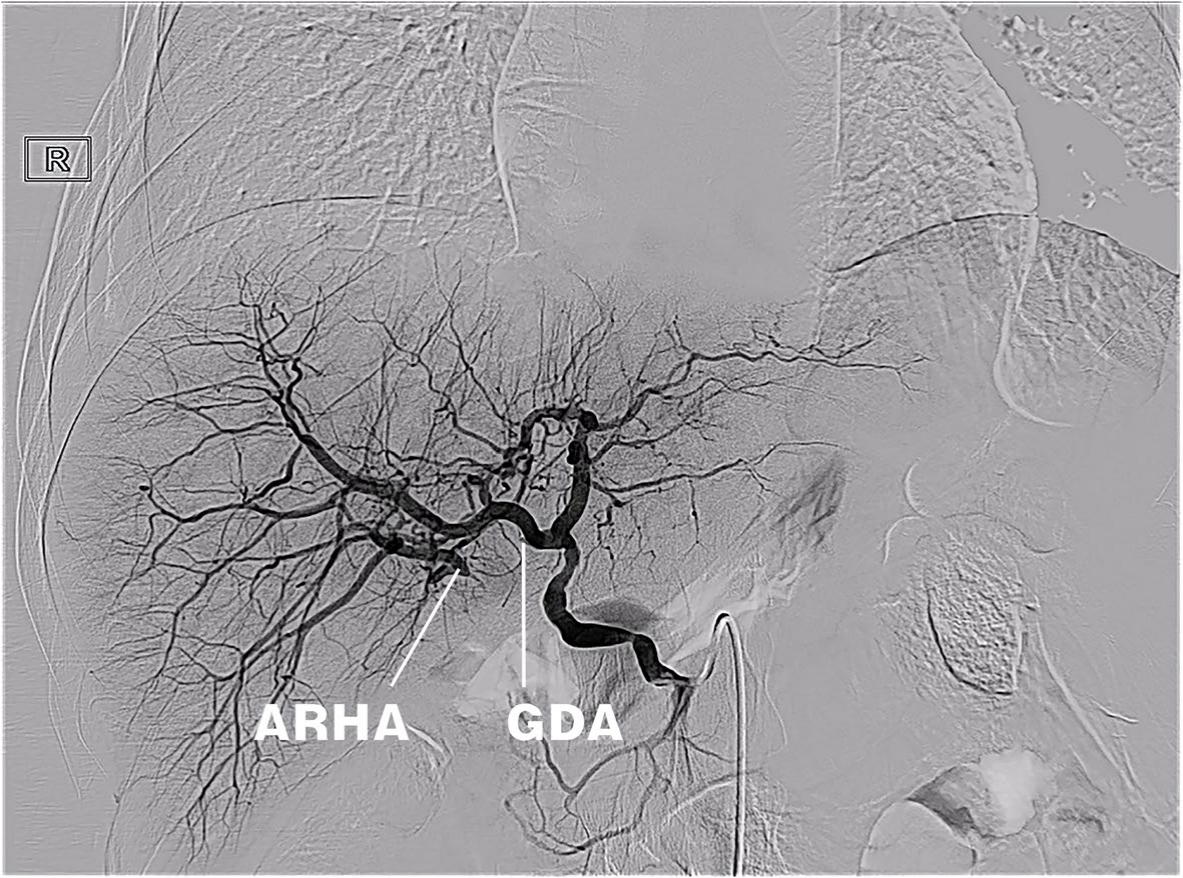
Anastomotic rupture of the accessory right hepatic artery and gastroduodenal artery. The accessory right hepatic artery and right hepatic artery had communicating branches in the liver

**Fig. 4:**
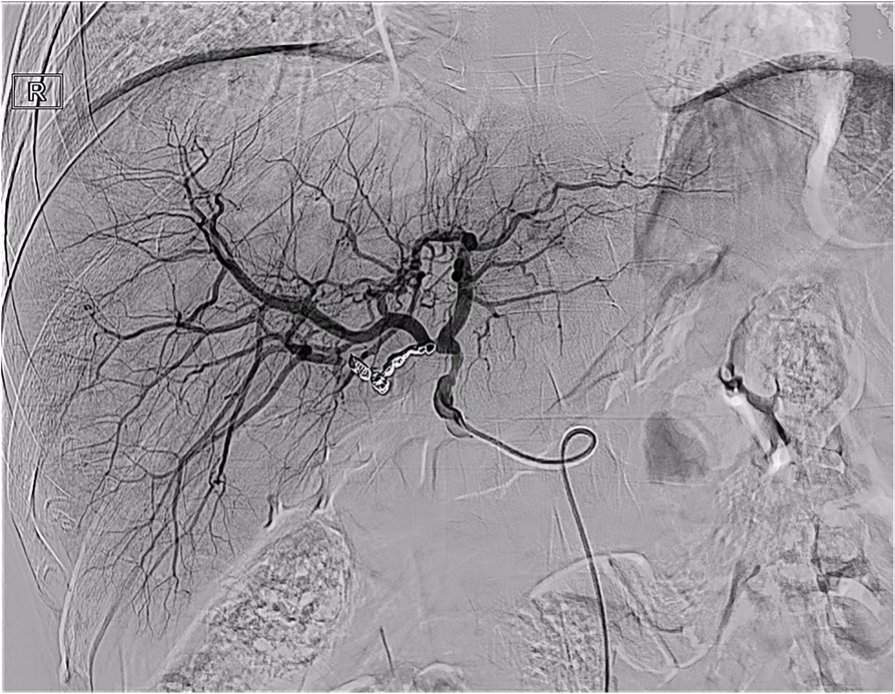
The distal end of the right accessory hepatic artery and the proximal end of the gastroduodenal artery were embolized with coils

On the 27^th^ day after LT, the T-tube intermittently drained red bloody fluid. Doppler ultrasound examination showed an area of no echo on the right side of the first hepatic hilum (range of approximately 1.3 × 1.0 cm). Hepatic arteriography showed that the LHA, RHA, and their branches were well developed. The anastomotic stoma of the accessory right HA had ruptured, but the distal trunk and branches were well developed, and there were communicating branches between the accessory right HA and RHA (Fig. 3). Selective HA embolization was performed by interventional therapy. The proximal and distal ends of the anastomotic stoma of the accessory right HA were embolized with coils (Fig. 4).

**Fig. 5:**
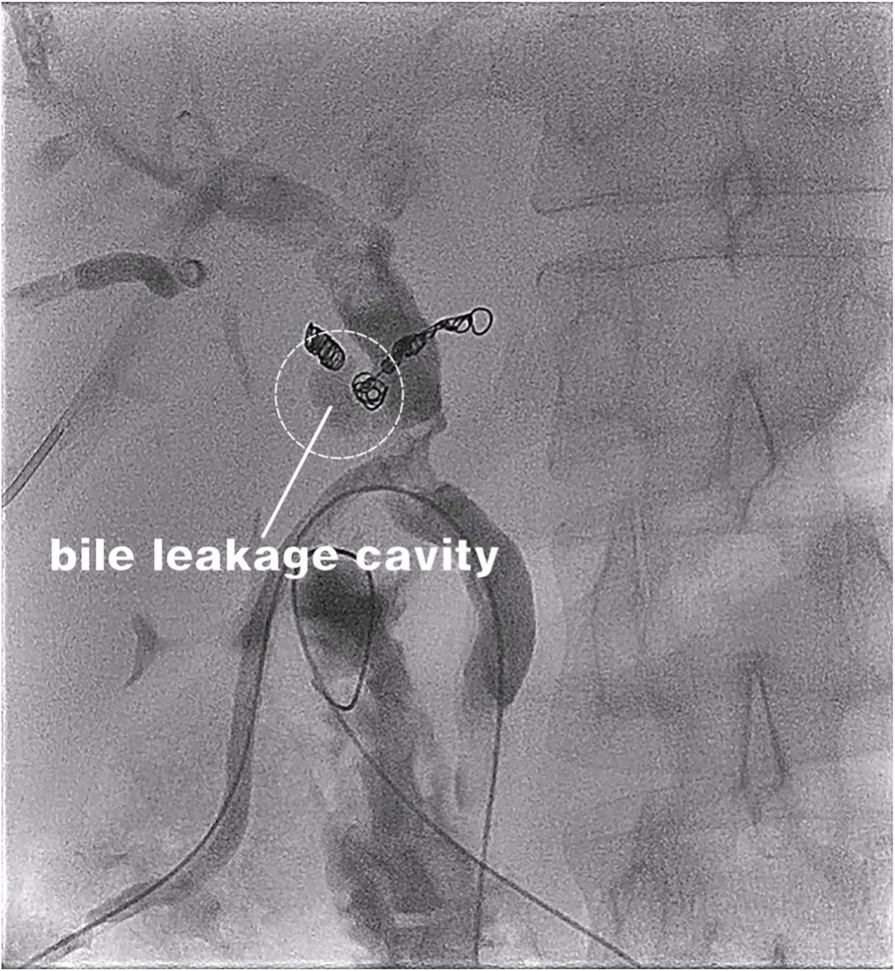
A cavity with overflow of contrast medium can be seen on the right side of the bile duct anastomosis and around the anastomosis of the original accessory right hepatic artery and gastroduodenal artery

**Fig. 6:**
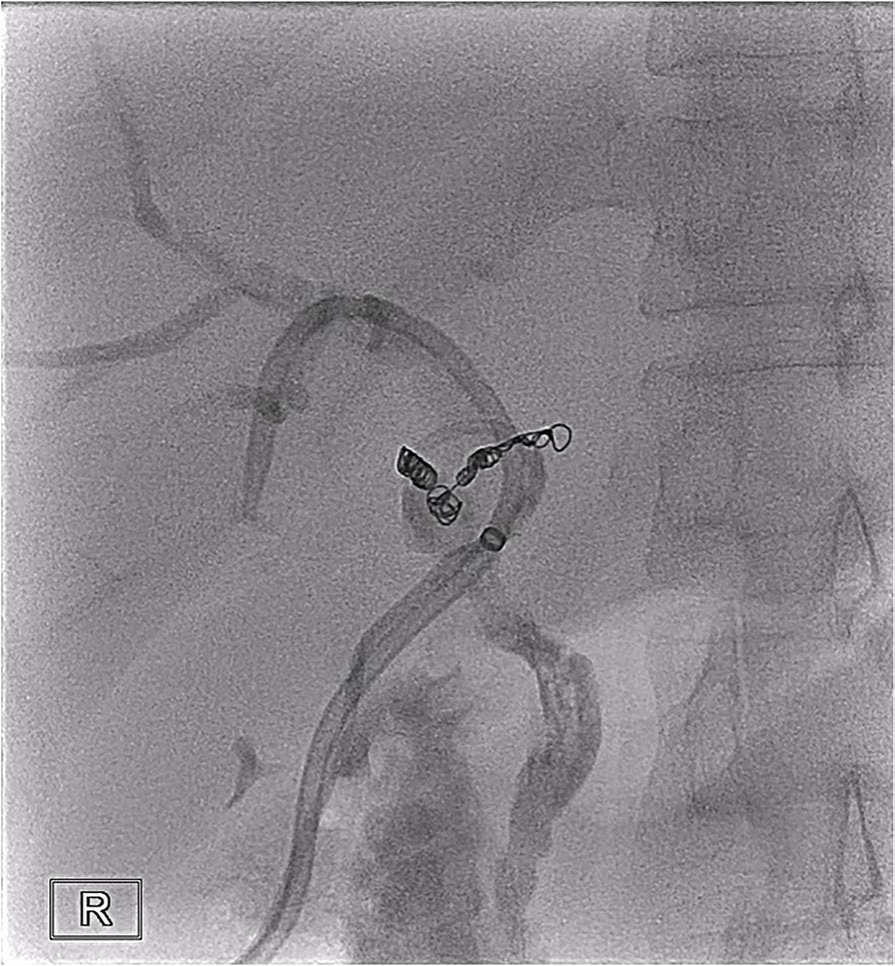
The drainage tube was placed into the intrahepatic bile duct and bile leakage cavity via the bile duct sinus

**Fig. 7:**
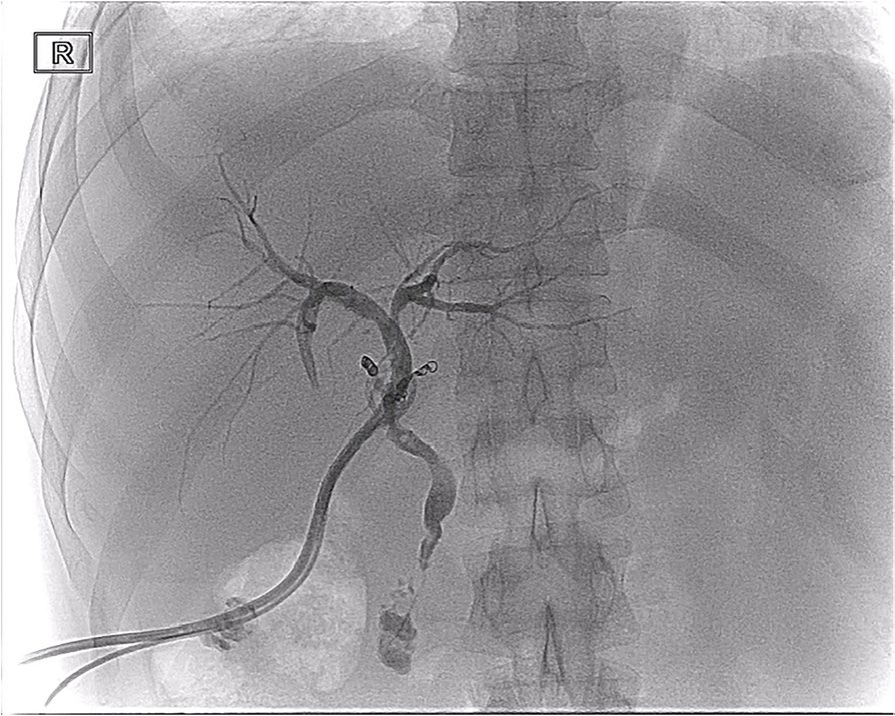
T-tube cholangiography showed that the bile leakage cavity around the bile duct anastomosis was significantly smaller than at previous imaging (March 17, 2021 vs February 24, 2021)

**Fig. 8:**
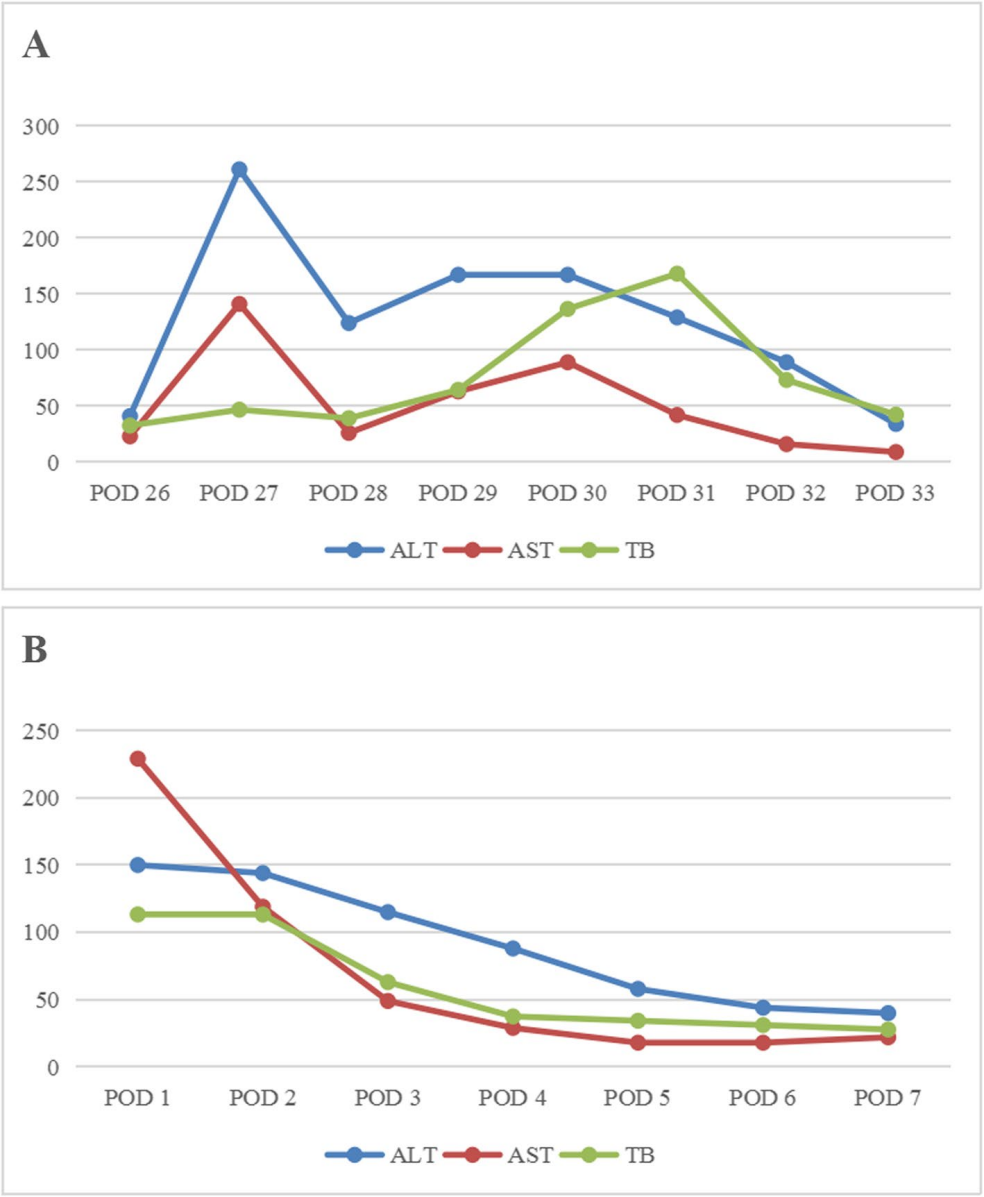
**A** Trends in alanine aminotransferase (ALT), aspartate aminotransferase (AST), and total bilirubin (TB) after arterial complication in patient 2. **B** Trends in ALT, AST, and TB after OLT in patient 5

T-tube cholangiography showed that the contrast medium flowed around the anastomosis of the accessory right HA through the rupture of bile duct anastomosis (Fig. 5). We considered that the rupture and bleeding of the anastomotic orifice of the accessory right HA was caused by bile leakage. A drainage tube was placed in the bile leakage cavity around the anastomotic stoma of the biliary tract, and a drainage tube support was placed in the biliary tract (Fig. 6). The residual bile leakage cavity was continuously flushed with normal saline. On March 17, 2021, T-tube cholangiography showed that the bile cavity around the bile duct anastomosis was significantly smaller than before (Fig. 7). Thereafter, liver function gradually returned to normal (Fig. 8A). There were no complications such as liver necrosis or infection.

### Patient 5

**Fig. 9:**
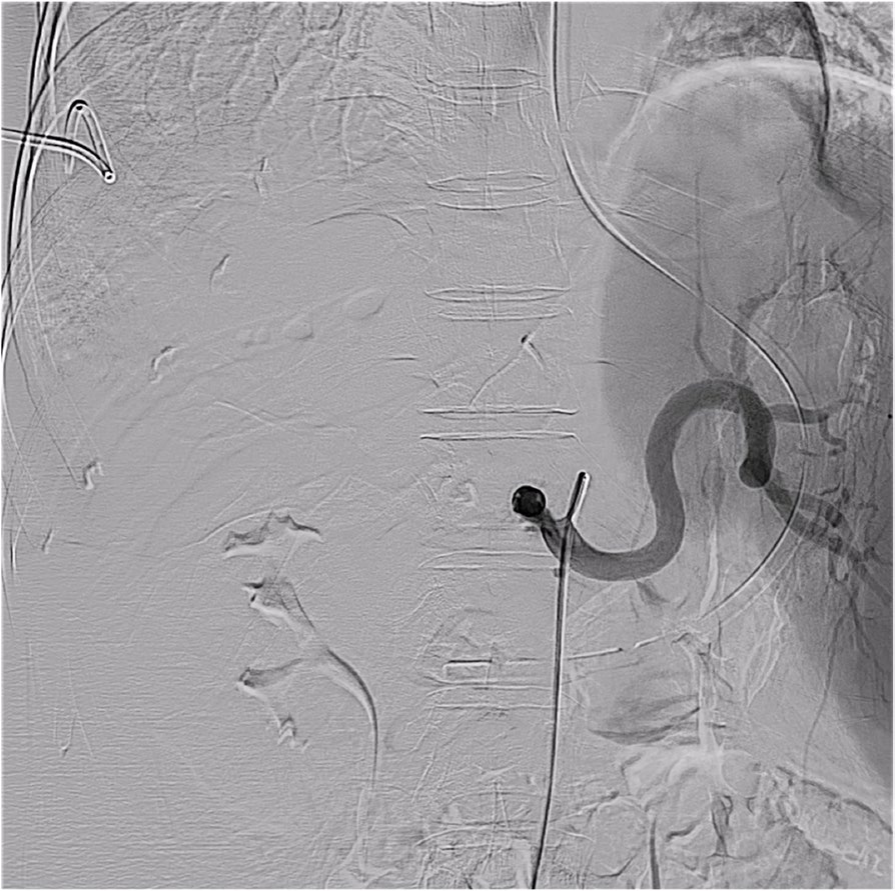
Arteriography showed arterial anastomotic thrombosis and splenic artery steal

**Fig. 10:**
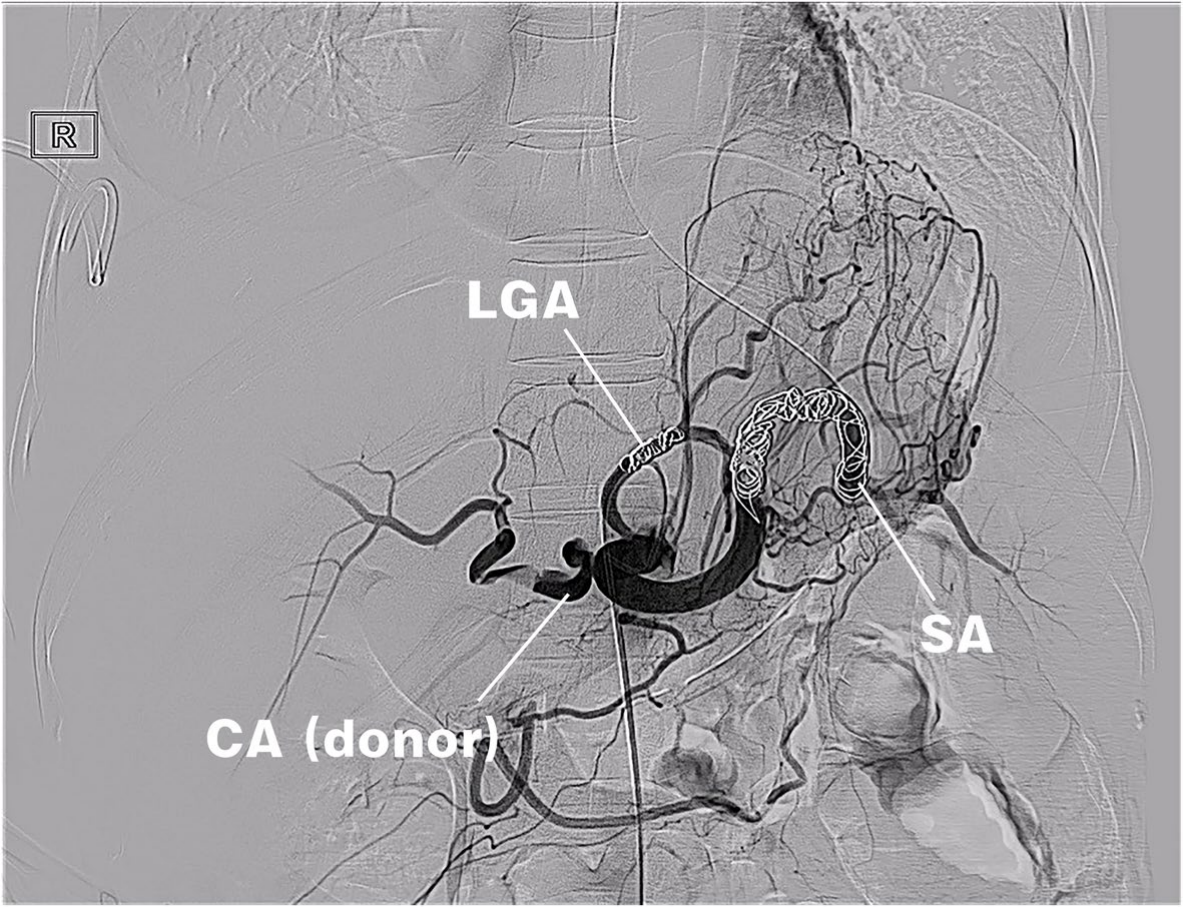
Hepatic blood supply was significantly improved after splenic artery and left gastric artery embolization

On the 3^rd^ day after LT, the HA was not observed on Doppler ultrasound. Hepatic arteriography showed thrombosis at the anastomotic site, no evidence of the HA and significant widening of the splenic artery (Fig. 9). We used coils to embolize the splenic artery and the LGA (Fig. 10), and administered urokinase thrombolysis via a microcatheter. Follow-up imaging displayed the HA, the LHA, and the RHA, while the anastomotic stoma of accessory left HA was blocked and the branches of accessory left HA were displayed in the liver. After interventional therapy, the patient was administered anticoagulant therapy. Color Doppler ultrasound showed normal blood flow of the HA and portal vein, while liver function gradually returned to normal (Fig. 8B). There were no complications such as liver necrosis or infection.

## Discussion

In OLT, the blood supply of the HA is very important for graft survival and prevention of biliary complications. Appropriate HA reconstruction is the key to success of OLT. Hepatic artery thrombosis (HAT) is a life-threatening complication in LT and may require retransplantation [2]. HAT presents in 3%–9% of all OLTs [3]. Although the risk factors for HAT have been identified, there are few systematic analyses of the role of HA variants and the reconstruction type on outcomes [4, 5]. The quality of arterial reconstruction is essential to prevent arterial thrombosis, which in turn leads to biliary complications and graft loss.

HA variation accounts for approximately 20% of HA anatomy [6, 7]. The most common HA variations include LHA originating from the LGA, an RHA arising from the superior mesenteric artery, or both. The reconstruction methods for LHAs and RHAs include preservation or ligation of abnormal HA branches, which depend on whether the LHAs and RHAs are replaced or attached. The Michels classification describes the differences between “an accessory” and “a replaced” HA. Accessory LHAs and RHAs were reported to be present in 3.2% and 1.6% of patients, respectively, and injury to these vessels was not associated with any major problems [8, 9]. It was previously reported that donor anatomical variations of HA, which require complex arterial reconstruction, may be associated with a higher incidence of arterial complications [10]. Schroering et al. [11] found that the HA length remains the shortest during HA reconstruction in liver transplantation, and all kinds of HA variants can achieve good long-term results. However, Karakoyun et al. found no overall differences in biliary and arterial complications or graft and patient survival between variant and standard arteries. Nevertheless, when the graft artery length is too long, the prognosis is worse [12]. In that study, the variant group had a longer arterial warm ischemia time and higher rate of preoperative arterial re-reconstruction.

HA complications such as thrombosis after LT have a significant effect on mortality and morbidity. Common complications involving the HA after LT include HAT, HA stenosis, HA pseudoaneurysm and its rupture, and arteriovenous fistula [13]. Hypothetically, the decision to interrupt the arterial blood flow of an AHA could result in dangerous complications such as HAT, primary graft non-function, early allograft dysfunction, and biliary stenosis or leakage [14]. By contrast, sparing an HA involves the use of the coeliac trunk as the site of the single anastomosis, which can result in an excessively long graft artery or a size discrepancy between the two anastomosed vessels. Ma et al. [15] showed that appropriate plastic HA reconstruction based on the caliber of donor and recipient vessels before LT involves preparing a single anastomotic vessel and recipient artery anastomosis, preservation of the arterial blood supply of each part of the donor liver, and protection of the donor HA integrity, which is an important factor for reducing the incidence of HA complications after LT [16].

It is important to note that it is not always possible to determine whether an artery is replaced or an accessory because of intrahepatic arterial branches that are not dissected, while cholangiography is not performed routinely. Montalti et al. [17] evaluated the utility of five parameters for determining whether an ALHA is accessory or replaced. In that study, the diameter of the ALHA was > 3 mm, while ischemic liver demarcation and absence of a Doppler arterial signal was observed in the left lobe after reconstruction of the common HA blood flow. In these cases, the ALHA was a replaced artery. Evidence of backflow from the GDA at the back table or evidence of arterial backflow from the ALHA after arterial reperfusion, the ALHA was an accessory artery in these cases.

Yilmaz et al. [18] reported that a better anatomical fit can be achieved in anastomoses performed after arterial perfusion, while redundancy or malrotations can be prevented when the ARHA of the graft is anastomosed to the graft’s GDA in an end-to-end fashion. For the right lobe, it is beneficial to anastomose both double arteries [19]. However, satisfactory arterial pulsatile backflow from the other artery is possible after dominant artery anastomosis in the right lobe. In this situation, anastomosis of the second artery is not required. Nevertheless, despite the pulsatile backflow from the smaller artery after the dominant HA anastomosis, cases of biliary ischemia in the relevant segment have been reported [20].

Arterial complications are known to be related to HA variations, ischemia–reperfusion injury, surgical factors, and perioperative management. Whether the ALHAs or ARHAs are retained depends on the specific situation during the operation. In practice, LT surgeons often struggle to preserve the integrity of the HA. The accessory HAs of the donor liver were preserved and anastomosed in the seven patients in the present study, while complications related to arterial anastomosis occurred in two of these patients. Patient 2 exhibited an accessory right HA. To completely preserve the blood supply of the liver, the GDA of the donor was anastomosed with the accessory right HA of the donor, resulting in anastomotic rupture and bleeding caused by bile leakage after surgery. We considered that if the accessory right HA was directly ligated rather than anastomosed, there would be fewer arterial anastomoses and reduced chance of bleeding. Patient 5 in the present study received a donor liver with an accessory left HA from the LGA. We choose to retain the accessory left HA, and the celiac artery of the donor liver was anastomosed with the bifurcation of the proper HA and GDA of the recipient. This caused the anastomotic artery to be too long to distort, and anastomotic thrombosis occurred after surgery. The HA was then recanalized via interventional therapy. However, the accessory left HA remained blocked. In both of these patients the accessory HA was occluded, although there were no complications such as biliary stricture, liver necrosis, or infection.

In most cases, the AHA has communicating branches with the internal HA [17]. Our center believes that preservation of the AHA can increase the number of arterial anastomoses and result in arteries that are too long or even angled. The requirement for AHA reconstruction and excessive anastomosis (i.e., kinking) length are the two main surgical risk factors for development of HAT. In living donor liver transplantation (LDLT), single HAR in left lobe with two arterial stumps did not affect patient survival or the incidence of biliary complications [21]. The research of Yilmaz S et al. [22] suggests that chose to anastomose both arteries if possible in liver grafts with double arteries, although nastomosing only 1 graft artery is not a risk factor for graft failure in LDLT. Multiple anastomoses result in a longer warm ischemia time and more surgical sutures—these factors have been reported as independent risk factors for the development of HAT in several studies [10].

## Conclusion

We suggest that AHAs determined as accessory arteries should be ligated as necessary. This can reduce the incidence of arterial complications, contribute to the perioperative management of LT patients, and improve the prognosis of LT. Note that a limitation of our study was the small sample size. At present, there is no unified opinion on whether the accessory HAs require reconstruction. Thus, future multi-center, large-sample clinical studies are required to validate our results and to evaluate the outcome of ligation of accessory HAs.

## Data Availability

All data generated or analyzed during this study are included in this published article.

## Abbreviations

AHA: Aberrant hepatic artery
ALHA: Aberrant left hepatic artery
ALT: Alanine aminotransferase
ARHA: Aberrant right hepatic artery
AST: Aspartate aminotransferase
CA: Celiac artery
CHA: Common hepatic artery
GDA: Gastroduodenal artery
HA: Hepatic artery
HAS: Hepatic artery stenosis
HBV: Hepatitis B virus
LDLT: Living donor liver transplantation
LGA: Left gastric artery
LHA: Left hepatic artery
LT: Liver transplantation
MELD: Model for end-stage liver disease
OLT: Orthotopic liver transplantation
PBC: Primary biliary cirrhosis
PVT: Portal vein thrombosis
RHA: Right hepatic artery
SA: Splenic artery
SMA: Superior mesenteric artery
TB: Total bilirubin
